# Minimizing AED Adverse Effects: Improving Quality of Life in the Interictal State in Epilepsy Care

**DOI:** 10.2174/157015909788848857

**Published:** 2009-06

**Authors:** EK St. Louis

**Affiliations:** Mayo Clinic, Rochester, Minnesota, USA

**Keywords:** Epilepsy, antiepileptic drugs, adverse effects, quality of life, interictal state.

## Abstract

The goals of epilepsy therapy are to achieve seizure freedom while minimizing adverse effects of treatment. However, producing seizure-freedom is often overemphasized, at the expense of inducing adverse effects of treatment. All antiepileptic drugs (AEDs) have the potential to cause dose-related, “neurotoxic” adverse effects (i.e., drowsiness, fatigue, dizziness, blurry vision, and incoordination). Such adverse effects are common, especially when initiating AED therapy and with polytherapy. Dose-related adverse effects may be obviated in most patients by dose reduction of monotherapy, reduction or elimination of polytherapy, or substituting for a better tolerated AED. Additionally, all older and several newer AEDs have idiosyncratic adverse effects which usually require withdrawal in an affected patient, including serious rash (i.e., Stevens-Johnson Syndrome, toxic epidermal necrolysis), hematologic dyscrasias, hepatotoxicity, teratogenesis in women of child bearing potential, bone density loss, neuropathy, and severe gingival hyperplasia. Unfortunately, occurrence of idiosyncratic AED adverse effects cannot be predicted or, in most cases, prevented in susceptible patients. This article reviews a practical approach for the definition and identification of adverse effects of epilepsy therapies, and reviews the literature demonstrating that adverse effects result in detrimental quality of life in epilepsy patients. Strategies for minimizing AED adverse effects by reduction or elimination of AED polytherapy, appropriately employing drugsparing therapies, and optimally administering AEDs are outlined, including tenets of AED selection, titration, therapeutic AED laboratory monitoring, and avoidance of chronic idiosyncratic adverse effects.

## QOL: A NEW TREATMENT PARADIGM IN EPILEPSY

Although analysis of quality of life (QOL) in epilepsy began later than many other medical fields, an ever growing body of epilepsy QOL research has accumulated over the last decade. A review of all the validated instruments for research and clinical use in epilepsy is beyond the scope of this article; two excellent recent reviews addressing the range of available tools and their relative advantages and potential uses are available for the interested reader [16,28]. In general, QOL research emphasizes measures of a patient’s general wellness in a particular disease state, incorporating a multidimensional health assessment of physical, psychological, and social domains affected by the illness and its treatment; these domains comprise most commonly utilized instruments in epilepsy research and practice, the QOLIE-89, QOLIE-31, and QOLIE-10 [28].

Epilepsy QOL research has re-emphasized the importance of producing seizure-freedom as a principle goal for all patients, but has also revealed that the interictal state is a principle determinant of a patient’s own perceived quality of life; in addition to seizures themselves, a patient’s daily functioning, cognitive status, mood states, social functioning, and the closely related factor of perceived adverse treatment effects determine how a patient feels about their overall QOL. QOL research in epilepsy has reshaped priorities in epilepsy care, calling for clinicians to maintain vigilance over the patient’s interictal status in the monitoring of mood, cognition, social functioning, and adverse effects in addition to seizure burden.

## DEFINING AND IDENTIFYING ADVERSE EFFECTS OF EPILEPSY THERAPIES

An adverse effect of therapy may be defined as any clinical symptom, sign, or laboratory dyscrasia which is deemed undesirable to the patient, the physician, or both. Adverse effects, while always unintended, are not entirely unexpected in epilepsy care; adverse effects are unfortunately common, reported in 40-50% of epilepsy patients receiving monotherapy AED treatment [2,21]. Most common are dose-related adverse effects, sometimes termed “neurotoxic” adverse effects, which may include (but are certainly not limited to) drowsiness, dizziness, fatigue, headache, blurry or double vision, impaired concentration or memory, or incoordination. Some AEDs are apt to produce certain characteristic adverse effects. A list of AEDs and common adverse effects (which is by no means exhaustive or comprehensive) is given in Table **1**.

Identifying AED toxicity in affected patients is problematic for several reasons. Some patients may have grown used to feeling ill while receiving AED therapy and complacently accept adverse effects as a natural consequence of their treatment. Additionally, patients are often understandably reluctant to “make waves” during clinic visits. Patients’ fears of ongoing seizures tend to overshadow complaints about medication toxicity, and conversely, seizure-free patients are often too comfortable with and grateful for their outcomes to broach the risk of lowering or withdrawing AEDs producing adverse effects, despite limitations in QOL.

Furthermore, patient reticence to raise concerns over toxicity is compounded by physicians’ preoccupation with assessment of seizure events (perhaps driven in part by overly busy clinics which discourage thorough communication), leading physicians to focus only on seizure histories while neglecting to inquire about AED toxicity and other important related interictal factors. Physicians also typically place greater concern about serious idiosyncratic adverse effects despite their infrequency due to fear of litigation; instead of being vigilant to bedside measures of common adverse effects, physicians may instead too often focus on clinical laboratory values such as AED blood levels, chemistries, liver function testing, and complete blood counts. However, while a valuable adjunct to guide clinical decision-making, AED blood levels are neither a fail-safe for detecting clinical toxicity or ensuring therapeutic seizure-freedom, and uncertainty exists as to the interpretation and clinical value of blood levels for many of the newer AEDs.

The availability of a rapid, efficient, and accurate tool for identifying common, dose-related, potentially remediable adverse effects in the office setting would greatly assist clinicians and patients alike. Fortunately, such a tool exists: the Adverse Event Profile (Table **2**) [2,3].

## AED ADVERSE EFFECTS ARE A PRINCIPLE DETERMINANT OF QOLIE

Antiepileptic drug adverse effects are a principle determinant of QOL in epilepsy patients. Proactive, quantitative assessment of adverse effects enables their identification and appropriate alteration of AEDs to reduce adverse effects. A recent randomized controlled trial of a rapid diagnostic tool for identification and quantification of AED adverse effects in a clinical office setting has been conducted, which evaluated the self-completed Adverse Event Profile (AEP, Table **2**) questionnaire [11]. This study convincingly demonstrated that clinicians who had access to information from the AEP more frequently adjusted their patients’ AEDs, resulting in improved patient quality of life, without sacrificing seizure control. The authors concluded that routine use of the AEP in epilepsy clinics may aid clinicians in identifying adverse effects of AED therapy, and consequently guide them in adjusting the AED regimen appropriately to obviate adverse effects. This research suggests that the clinicians’ well intentioned quest to produce seizure-freedom often leads to overtreatment with AEDs; pushing AED doses ever upward, and adding AED upon AED in a spiraling fashion may have clear negative consequences for quality of life.

## MINIMIZING AED ADVERSE EFFECTS

AEDs should be adjusted to achieve the clinical goals of seizure-freedom without adverse effects. This may be a delicate balancing act for some patients, since all AEDs have the potential to cause dose-related, “neurotoxic” adverse effects. Fortunately, adverse effects may be obviated in most patients by dose reduction, reducing or eliminating AED polytherapy, or substituting for a better tolerated AED.

One relatively simple solution to avoiding or minimizing AED adverse effects is a class switch from an older to a newer AED in patients who are experiencing toxicity on their current therapy, and selecting newer AEDs for new onset epilepsy patients who may be vulnerable to the development of AED toxicity. Older AEDs result in adverse effects of treatment in nearly half of patients [16,28]. Fortunately, the availability of several newer AEDs offers improved tolerability and safety profiles, helpful qualities that the clinician may employ to minimize adverse effects in epilepsy care. Since 1990, eleven new antiepileptic drugs rather than nine (AEDs; felbamate, gabapentin, lacosamide, lamotrigine, levetiracetam, oxcarbazepine, pregabalin, rufinamide, tiagabine, topiramate, and zonisamide) have been approved by the Food and Drug Administration (FDA) based initially on adjunctive therapy pivotal trial designs.

Several controlled trials have suggested that newer AEDs possess superior tolerability to older AEDs, especially in patient populations with specific vulnerability to AED toxicity such as the elderly [5,32]. Unfortunately, higher cost continues to limit access of newer AEDs for many patients, but availability of reliable generic formulations of many newer AEDs has increased recently, which could largely obviate this barrier.

Another principle for avoiding and minimizing adverse effects is to employ monotherapy at the lowest effective dose whenever possible, and reserve AED polytherapy at an acceptable total drug load for refractory epilepsy. While all older AEDs were “grandfathered” in their indication for monotherapy treatment of epilepsy by FDA on the basis of comparator trial data and longstanding clinical experience, monotherapy approval and data supporting use for most newer AEDs is more limited. Gabapentin, oxcarbazepine, and lamotrigine possess randomized controlled trial evidence for monotherapy treatment of partial-onset seizures and topiramate has evidence for monotherapy use in new onset epilepsy [9]. Although felbamate possess monotherapy efficacy data, its sole use currently is in brittlely refractory epilepsy, given its risk of idiosyncratic toxicities.

## REDUCING POLYTHERAPY REDUCES ADVERSE EFFECTS

AED polytherapy should be reserved for use in refractory epilepsy. When polytherapy is necessary to achieve seizure control, utilizing the lowest possible drug load (the lowest numbers and doses of AEDs) is desirable. Many medically refractory epilepsy patients require chronic polytherapy, and the recent AAN/AES Practice Guidelines for the treatment of refractory epilepsy stated that all newer FDA-approved AEDs have Class 1 evidence for adjunctive treatment of refractory partial-onset seizures in adults, and there is ample evidence to conclude that lamotrigine, oxcarbazepine, and topiramate are effective for the treatment of refractory partial seizures in children [10]. While no good evidence for specific AED polytherapy combinations exists, augmenting monotherapy with an AED offering a different or complementary mechanism of action may be considered.

Eleven new antiepileptic drugs rather than nine. When polytherapy is prescribed, great care must be taken to avoid excessive drug dosing and drug-drug interactions that may increase clinical toxicity through pharmacokinetic factors. Considerable research has demonstrated that the most deleterious result of polytherapy is an increased risk for development of adverse effects, including pharmacodynamic dose-related neurotoxic effects, severe life threatening rash with combined lamotrigine and valproate, and teratogenic effects in children born to pregnant women receiving AED polytherapy. A recent survey of patients with epilepsy demonstrated that central nervous system-related adverse effects, especially memory complaints and fatigue, appear to be most frequent overall, and that polytherapy was more frequently associated with these adverse effects than monotherapy [6].

Unfortunately, only a small number of refractory patients can be rendered seizure-free with AED polytherapy, although some patients benefit substantially by reduction of seizure burden. A recent pivotal prospective observational study demonstrated that only 3% of patients who fail two sequential initial monotherapies are subsequently rendered seizure-free by polytherapy [13]. Since AED polytherapy only rarely results in seizure freedom, the overall philosophy of AED therapy for refractory epilepsy necessarily shifts from effecting seizure-freedom to the more modest goal of seizure palliation. While no epilepsy care should be rendered with a defeatist attitude, a practical realization that the refractory patient is highly unlikely to benefit from overly aggressive AED dosing may prevent overtreatment of the patient with ever escalating doses and numbers of AEDs in a polytherapy regimen. Instead, establishing that a patient has refractory epilepsy suggests the need for proactive exploration of non-pharmacologic treatment alternatives to AED therapy that offer the potential benefits of reducing AED drug load and minimizing adverse effects.

## DRUG SPARING THERAPIES MAY ENABLE AED REDUCTION

Failure to produce seizure control after even one well-tolerated, optimally administered monotherapy AED is an ominous prognostic feature that may portend medical intractability in many patients [14]. After failure of one or two AED monotherapies, patients with epilepsy should receive strong consideration of additional diagnostic evaluation for non-pharmacological therapies, including state-of-the art neuroimaging to detect lesional epileptogenic pathology that is potentially amenable to surgical resection, and ictal video-electroencephalography (vEEG) for definitive epilepsy syndrome classification and possible localization. Potentially “drug sparing” non-pharmacologic therapies may afford opportunities to reduce AED adverse effects by minimizing AED drug numbers and dosages, thereby improving QOL. Currently available “drug-sparing” therapies include epilepsy surgery, the vagus nerve stimulator, dietary therapies, and exploration of co-morbid primary sleep disorders that may aggravate epilepsy. However, each of these therapies have their own unique set of potential adverse effects that are quite different from those of the AEDs to consider.

The superiority of epilepsy surgery over medical therapy for well-localized mesial temporal lobe epilepsy, the most common refractory partial epilepsy syndrome, has been well established in a randomized controlled trial [38]. Patients with mesial temporal lobe epilepsy were randomized to anterior temporal lobectomy surgery or best medical therapy. At one-year, an intention to treat analysis demonstrated superiority of surgery; 58% of patients randomized to surgical therapy were rendered seizure-free (which rose to 64% of those who received surgery), whereas only 8% of patients became seizure-free on medical management. Patent QOL was also improved significantly in the surgical group compared with the medical group, although the persistence of this difference weaned by the completion of the trial. A recent metaanalysis yielded similar data on seizure-control for temporal lobe resections, but suggested less robust outcomes varying between 27-46% patients rendered seizure-free following extratemporal resections [8,37]. Importantly, numerous studies have shown decreases in patient drug load when comparing pre-operative to post-operative dosages and numbers of AEDs. Adverse effects of epilepsy surgery include a minor risk (between 1-5% in major series) of major acute surgical complications such as hemorrhage, infarction, or infection, or of memory, language, visual, motor, or somatosensory functional loss depending upon the locali-zation of the surgical epileptic focus. Some patients with refractory partial epilepsy are not suitable epilepsy surgical candidates due to diffuse or unlocalizable epileptic foci, while others may have epileptic foci overlapping critical cerebral functional cortex. Still others may choose not to undergo brain surgery despite suitable candidacy. In these cases, other options may still exist.

The vagus nerve stimulator (VNS) is a FDA approved device as an adjunctive treatment for refractory partial-onset onset seizures. Long-Term follow-up outcome data suggests that over 40% of patients experience a 50% or greater reduction in their seizures [24]. More recent evidence suggests that up to 15% of patients may become seizure-free when the device is implanted in patients with milder refractory epilepsy (epilepsy duration less than 5 years, having failed 4 or fewer previous AED trials) [31]. There is no current evidence based guideline for the best timing of VNS placement, but most epileptologists reserve VNS for patients who are not resective surgery candidates or who refuse surgery, and for patients who have filled several older and new AEDs, given that seizure free efficacy is less with VNS than with resection surgery for carefully selected well-localized patients, and generally efficacy of VNS to that seen with AEDs. However, VNS offers additional benefits to seizure burden reduction alone in many patients; VNS therapy has been noted to improve patient QOL, possibly by improving important determinants of QOL in epilepsy such as alertness, mood and memory which may be due in part to successful reduction of AED loads [7,15]. A distinguishing advantage of VNS therapy is its lack of sedating or neurotoxic adverse effects; instead, VNS adverse effects such as throat discomfort or vocal hoarseness are usually mild, temporary, and related only to the stimulation-on duty cycle, and VNS adverse effects are usually easily mitigated by alterations in stimulation parameter settings. Predictors of which precise epilepsy localizations and etiologies are most likely to benefit from VNS, and the optimal dosing of the device once it has been implanted, are yet to be defined in prospective clinical trials.

Specialized diets may be a useful adjunctive treatment for epilepsy. The best studied of these is the ketogenic diet. The ketogenic diet is a high fat, low protein, low carbohydrate diet that induces systemic ketosis, which has an antiepileptogenic effect on the brain. The ketogenic diet is most often successfully employed in children, but may also be tried in adolescents and adults. Unfortunately, unless rigid compliance is assured, the ketogenic diet produces little benefit and, in general, most adolescents and adults have limited tolerance of the diet. However, highly motivated and desperately refractory epilepsy patients may benefit from the ketogenic diet. An alternative that is often more tolerable, but not yet robustly studied, is the modified Atkins diet, a high fat, moderate protein, low carbohydrate diet that induces mild ketosis. The risk of inducing undesirable lipid abnormalities, metabolic states, and other long-term health effects of these dietary therapies also remain unclear.

Identifying and treating seizure aggravators is also an important consideration. Recent studies have suggested that obstructive sleep apnea (OSA) is a frequent co-morbidity in refractory epilepsy, and a pilot treatment trial of nasal continuous positive airway pressure (nCPAP) in patients with refractory epilepsy and co-morbid OSA showed substantial seizure reduction without alteration of AED therapy [17-19]. Primary sleep disorders such as OSA, restless legs syndrome, and periodic limb movements of sleep may fragment sleep and worsen seizure burden in patients with refractory epilepsy. If a primary sleep disorder is suspected, a diagnostic polysomnogram should be ordered, and aggressive treatment for any primary sleep disorder discovered should be initiated. One limitation of therapy for OSA is that adverse effects and poor tolerability of nCPAP therapy are common; tolerability and potential risks of nCPAP therapy in those with co-morbid epilepsy have not been reported and merit prospective investigation.

## PRACTICAL STRATEGIES FOR OPTIMAL AED ADMINISTRATION TO MINIMIZE ADVERSE EFFECTS

Guidelines for choosing and optimally administering epilepsy therapies are currently lacking. Until evidence-based guidelines are developed, optimal selection and administration of epilepsy therapies must be highly individualized by synthesizing available data and patient qualities and preferences. AED selection, titration, target dosage, basic pharmacokinetic properties, potential AED and non-AED drug interactions, therapeutic AED laboratory monitoring, and avoidance of chronic idiosyncratic adverse effects are all considerations when attempting to minimize adverse effects of AED administration. There are currently no clear evidence based algorithms to guide these important factors in AED treatment. Nonetheless, common treatment principles underlie the choosing, dosing, sequencing, and monitoring of AED therapy in epilepsy care. While a comprehensive guide to employing AED therapy is beyond the scope of this article, several basic principles for optimal AED administration include:

- Choose AED therapy appropriate for the epilepsy syndrome;
- Consider patient characteristics and co-morbidities when choosing AEDs;
- Employ AED monotherapy at the lowest effective dosage to achieve seizure freedom;
- Reserve AED polytherapy (combining two or more AEDs) for refractory patients and minimize total drug load to limit adverse effects;
- Treat according to the patient’s clinical response, not the AED level;
- Monitor for long-term complications of older AED therapy and consider withdrawal of therapy when appropriate; and
- Choose affordable AED therapy.

A few of these points which are particularly important for avoiding and minimizing adverse effects are next considered.

## AED SELECTION

A first general principle in AED choice and usage is to employ AED monotherapy whenever possible, since monotherapy is just as effective, or more effective, than polytherapy. Monotherapy limits the potential for adverse effects and drug interactions. Specific AEDs are itemized with accompanying information on clinical spectrum of uses, pharmacokinetics, typical dosing and blood levels, and cardinal adverse effects in Table **1**. When choosing and utilizing AEDs, a considerable amount of pharmacological knowledge and clinical wisdom is necessary for successful patient outcomes. Patient’s characteristics such as age, sex, co-morbidities, and co-existing medications are important determinants of proper drug selection and use. These factors most frequently guide which AED may have the most desirable pharmacokinetic and phamacodynamic properties to avoid the development of undesirable adverse effects, and examples of patient characteristics that impact on AED selection are now discussed.

Older patient age is a factor suggesting a heightened vulnerability to the development of dose-related neurotoxic adverse effects. Several recent studies have shown that newer AEDs are better tolerated in the elderly [5, 32]. Female sex is an important determinant of AED selection; women usually have lower bone density and may therefore be most vulnerable to development of osteopenia, and growing evidence now suggests that several older AEDs may accelerate bone loss. In women of childbearing potential, AEDs that are associated with teratogenicity and which may interact with hormonal contraceptives also merit cautious use, and may be best avoided. Patient co-morbidities may affect the choice of an AED. For example, weight is an important consideration. Valproate, pregabalin, and carbamazepine may contribute to weight gain, while topiramate and zonisamide may include weight loss among their “adverse effect” profile. The patient with both epilepsy and migraine might favor topiramate or valproate, drugs which are efficacious for both conditions. Patients receiving other AEDs or non-AED co-medications interacting with AEDs are at particular risk for adverse effects of treatment and adverse consequences. Hormonal contraceptive and anticoagulants are perhaps the best known non-AED medications resulting in hazardous interactions with enzyme-inducing AEDs (EIAEDs), but other inducible medications such as lipid-lowering drugs and anti-hypertensives may result in catastrophic cardiovascular and cerebrovascular consequences if rendered less effective by EIAEDs.

## AED TITRATION AND TARGET DOSE

AED dosing must be individualized to achieve optimal results. The usual strategy is to titrate the AED toward a target dose that has proven effective for most individuals in pivotal clinical studies and in subsequent clinical experience. Dose adjustment can then be made in the event of adverse drug reactions or recurrent seizures. If the endpoint of seizure freedom is preserved, maintaining a lower but clinically therapeutic AED dosage and level (if obtained) is entirely acceptable. If a patient continues to experience breakthrough seizures, raising the AED dose to the maximal dose tolerated is sometimes necessary, although recent evidence demonstrates that only a minority of patients become seizure-free when dosed above the usual population range, so a practical viewpoint of treatment futility should be realized when patients experience frequent breakthrough seizures despite adequate AED dosages [14]. Therapeutic change should be strongly considered when seizure-freedom is not maintained at AED doses which are effective for most patients.

## AED SEQUENCING AND POLYTHERAPY CONSIDERATIONS

Overlapping AEDs in transitional polytherapy (where the baseline AED is maintained at the current dose to limit breakthrough seizures, the newly added AED is titrated to a protective dose, then the original drug is tapered and discontinued) is the preferred method when introducing a new AED monotherapy. Abruptly stopping the existing AED increases the risk of seizures while introducing the new adjunctive AED too rapidly may induce adverse effects that taint the patient’s perception of what could otherwise be an effective therapy. Recently, an expert consensus panel was convened to address the issue of how best to convert between AED monotherapies when initial monotherapy fails due to lack of efficacy or tolerability, utilizing the Delphi model of determining consensus on strategies for best practice [33]. The experts agreed on a basic principle to taper an existing baseline AED only after a presumably efficacious dose of the newly planned adjunctive AED is reached. It was felt that application of this principle should be modified by occurrence of adverse effects possibly attributable to the existing drug, in which case earlier or more rapid tapering of the existing drug should be considered. The experts agreed that seizure-free patients benefit from slower tapering in smaller decrements than would be typical for patients with seizures not controlled by the existing AED.

Most patients should receive two sequential AED monotherapies with differing mechanisms of action prior to attempting chronic polytherapy. In general, a similar strategy to that agreed upon by the expert panel for monotherapy conversions should also be used when initiating addition of a second (or third) adjunctive AED for use as chronic polytherapy. A recent trial well illustrated the potential for increased toxicity with adjunctive AED therapy [25]. This randomized, perspective, adjunctive topiramate study design directly addressed which strategy for addressing adverse effects emerging during adjunctive AED titration was most efficacious and tolerable, by randomizing subjects to two treatment arms; (1) a “Flex Dose” titration group, in which investigators were permitted to reduce the baseline AED as needed to permit titration of adjunctive topiramate; and (2) a “Fixed Dose” titration group, where investigators were not permitted to adjust the baseline AED for titration-emergent adverse effects (similar to the restrictions required in most pivotal labeling studies for the newer AEDs as adjunctive therapy); if investigators needed to adjust the baseline co-therapy, the patient was exited from randomized treatment. The percentage of patients exiting randomized treatment due to adverse effects was the primary study endpoint. The “Flex Dose” group achieved higher target doses of adjunctive topiramate, while the “Fixed Dose” group had nearly twice as many subjects drop-out for intolerable adverse effects [25]. This data suggests that it may be best to reduce the baseline AED to minimize adverse effects emerging during the titration of a new adjunctive therapy, to enable an adequate therapeutic trial of the new adjunctive AED therapy. The adjunctive AED can be further increased as needed to achieve optimal therapeutic doses in transition toward a new monotherapy when the new adjunctive AED demonstrates a meaningful therapeutic effect [4].

When utilizing polytherapy, the clinician must be knowledgeable about the potential for pharmacokinetic and pharmacodynamic AED interactions, which influence the risk of developing adverse effects. While an exhaustive review of drug interactions is beyond the scope of this article, a few illustrative scenarios will suffice to make this important point. In general, the main pharmacokinetic interactions to consider in AED polytherapy are potential Cytochrome P450 metabolism competition, and protein binding and displacement. Co-administration of the enzyme-inducing AEDs (i.e., phenobarbital, phenytoin, carbamazepine) with inducible AEDs (such as lamotrigine, topiramate, or tiagabine) increases and hastens the metabolism of the inducible AED. In complex polytherapy regimens, reducing the dose of enzyme-inducing AEDs (such as carbamazepine, phenytoin, or the barbiturates) will “de-induce” the regimen, thereby increasing the serum concentrations of highly inducible AEDs (such as lamotrigine and topiramate), leading to optimized pharmacokinetics of the inducible AED and improved seizure control in some instances [1]. Conversely, when an inhibitor such as valproate is given with lamotrigine, there is a greater chance of serious rash than when lamotrigine is given with enzyme-inducing AEDs [23]. Two recent studies of vulnerable institutionalized patients well illustrate the complex pharmacokinetic issues that can arise in polytherapy. In these studies of elderly nursing home and multiply handicapped patients, common use of undesirable pharmacokinetic AED combinations was found, especially phenytoin/phenobarbital and phenytoin/valproate polytherapy, [12,20]. The interactions between these AEDs are bidirectional, complex, and variable, leading to unpredictable increases or decreases in drug concentrations, and in an institutionalized patient population with common co-morbid hypoalbuminemia, free phenytoin levels should be monitored to enable appropriate co-therapy adjustments to avoid toxicity. Switching to a regimen with less likelihood of complex interactions should be strongly considered in such instances. An extensive, superb review of AED drug interactions is found in the recent work by Patsalos and colleagues [26].

Pharmacodynamic adverse effects are especially difficult to avoid when using polytherapy, as dose-related neurotoxic and cognitive adverse effects are more prevalent in polytherapy. Cognitive impairments commonly accompany polytherapy and are often subtle and difficult to identify without specifically questioning the patient. While standard office assessment of cognition often shows minimal impact, detailed neuropsychological and electrophysiological measures often show impairments in attention, concentration, executive function, and memory in patients receiving AED therapy [22,29,34,35]. Some adverse effects such as sedation, cognitive impairments, gait disturbance, and hair changes are consistently underreported unless patients are specifically questioned about the presence or absence of these symptoms. Clinicians should consider routinely using adverse events screening instruments such as the AEP to identify AED adverse effects during office visits, especially for patients receiving polytherapy. Some AEDs have a greater tendency to cause pharmacodynamic adverse effects when co-administered with other AEDs; for example, there is a greater chance for adverse effects when utilizing topiramate as adjunctive therapy than when it is administered as monotherapy [27,30].

## AED THERAPEUTIC MONITORING: TREAT THE PATIENT, NOT THE LEVEL

An intimately related issue to clinical toxicity meriting discussion is “laboratory” toxicity, that is, when AED blood levels fall above established “normal” therapeutic ranges. Philosophies on the use of AED blood level monitoring differ, but most agree that blood levels should be considered only a guideline to treatment in most cases. AED levels should not be perceived as an absolute indication for altering AED dosing, divorced from clinical judgment of the patient’s seizure control or adverse effects. Blood level monitoring can help guide therapy, but so-called “therapeutic” levels are derived from treatment of populations. An individual patient may require a lower or higher intensity of AED therapy to achieve optimal results. In general, AED levels are most useful when testing a clinical hypothesis. “Routine” or scheduled levels should be generally discouraged, an exception being chronic phenytoin therapy in institutionalized patients (where “zero-order” kinetics from non-linear hepatic metabolism may lead to drug accumulation and toxicity). AED blood level monitoring is further reviewed in the next article in this series.

## PREVENTION OF IDIOSYNCRATIC ADVERSE EFFECTS OF AEDS

Unfortunately, accurate prediction of the development of idiosyncratic toxicities of AEDs is not currently possible. The highest risk of idiosyncratic reactions associated with most older AEDs such as serious rash, hepatotoxicity and hematologic dyscrasias is during the first 6-12 months of therapy, and extremely rare thereafter, so that routine monitoring for these problems is rarely necessary in otherwise healthy patients. Intermittent blood testing for monitoring of liver function tests and hematologic functions is reasonable, although not of proven value; consider obtaining baseline AST/SGOT and complete blood count (CBC) with repeated measures at 1, 3, and 6 months following initiation of therapy with older AEDs, and measure baseline serum sodium before initiation of carbamazepine and oxcarbazepine (which may result in hyponatremia) with repeat testing after two months, perhaps with earlier or more frequent testing in elderly patients or those receiving sodium-wasting diurectics. If felbamate is used, baseline and weekly liver function and CBC testing is indicated per package insert labeling. Most experts continue such extensive monitoring until at least several months of clinical and laboratory stability have been reached, given the potential for fatal aplastic anemia and hepatotoxicity, and then continue periodic monitoring thereafter.

There is mounting concern that patients on chronic therapy with older AEDs are at risk for bone density loss and fractures. Enzyme-inducing AEDs (EIAEDS: i.e., carbamazepine, phenytoin, phenobarbital, primidone, oxcarbazepine, and possibly high dose topiramate) have the potential to decrease bone density through secondary hypoparathyroidism and decreased Vitamin D levels, and some evidence suggests that non-inducers such as valproate also lead to decreased bone density [36]. Many epileptologists are now proactively counseling patients who have received therapy with older AEDs for several years about the emerging risk of reduced bone density, suggesting measuring bone mineral densitometry, and placing patients on supplemental calcium. Offering patients the opportunity to attempt withdrawal from older AEDs if they have been seizure free for several years (and if they are at low risk of seizure recurrence), or transitioning them to a newer AED lacking enzyme-inducing pro-perties (although there is little evidence of long-term safety for bone health with newer AEDs) may also be considered.

Women of child bearing potential (WCBP) are at risk for two worrisome AED-related adverse effects: pregnancy due to oral contraceptive failure, and AED-induced fetal teratogenesis. Avoiding EIAEDs in WCBP whenever possible is prudent to avoid hormonal contraceptive failure, and counseling WCBP about the hazard of contraceptive failure with EIAEDs is important. WCBP should be instructed to utilize double-barrier contraception in addition to their hormonal methods when receiving EIAEDs. Prior to a planned pregnancy, weaning the WCBP from AEDs when she is seizure-free and at low risk of seizure recurrence, or utilizing monotherapy when AED therapy is necessary whenever feasible, is especially important in WCBP since polytherapy use has been linked to teratogenesis. While there is not yet unequivocal evidence regarding AED safety or hazards during first trimester exposure in pregnancy, available evidence suggests that phenobarbital and valproate should be avoided when possible (unless these agents have resulted in complete seizure-freedom, in which case many experts still agree that the most desirable AED to maintain during pregnancy is the AED which has resulted in seizure control). Although no certain evidence basis exists. most agree that WCBP taking AEDs should receive folic acid 1 milligram daily (or a prenatal multivitamin), especially those on EIAEDs. Some experts recommend high dose folic acid for women receiving valproate, up to 4 milligrams daily.

Chronic phenytoin exposure is of particular concern, given its relatively common association with cosmetic adverse effects including gingival hyperplasia (which may be severe enough to warrant repeated gingivectomies), and the rare but real potential of axonal peripheral neuropathy and irreversible cerebellar ataxia. Many experts now feel that because of these risks, phenytoin use should be for a relatively short term (i.e., no longer than a few years), after which time the patient can be offered the opportunity to transition to another AED if continued therapy is necessary.

One additional idiosyncratic toxicity meriting mention here is risk of serious rash with lamotrigine, which appears to be related to several factors, at least one of which is modifiable: pediatric age, concurrent valproic acid therapy, and overly rapid initial titration of lamotrigine. When initiating lamotrigine, paying strict attention to AED co-medications and adhering strictly to the recommended package insert titration instructions is required.

## CONCLUSIONS

Epilepsy QOL research suggests that too little clinician attention has been focused upon the interictal state. One critical determinant of QOL in epilepsy patients is whether they suffer from adverse effects of AED therapy. Research has shown that identification of AED adverse effects is aided by using the Adverse Events Profile (AEP), a validated clinical tool easily completed by patients in the office setting within a few minutes, and that considering this information leads to appropriate alterations in AED therapy that may improve patient QOL. Strategies for reducing common dose-related “neurotoxic” adverse effects include optimal AED selection, titration, co-therapy reduction, utilizing monotherapy, and elimination of polytherapy when feasible. In refractory epilepsy, utilizing drug-sparing therapies such as epilepsy surgery, VNS or dietary therapies, or diagnosis and treatment of co-morbid primary sleep disorders may minimize AED doses and drug load, thereby minimizing adverse effects. Idiosyncratic adverse effects cannot be reliably predicted with current clinical tools and strategies for prevention of specific idiosyncratic adverse effects such as teratogenicity and bone loss are unfortunately lacking, but treating women of child bearing potential receiving AED therapy with folic acid and daily supplemental calcium is reasonable (the latter should also be considered even for men receiving chronic AEDs with risk factors for osteoporosis). Vigilance toward the current presence and future possible evolution of adverse effects of epilepsy therapy should be considered a chief responsibility of epilepsy clinicians, since proactive AED alteration may effect improvements in patient QOL.

## Figures and Tables

**Table 1:** Properties of the AEDs

Older AEDs	Spectrum of Effect	Daily Adult Dosage/Interval	Levels (uG/mL)	Usual Adverse Effects	Severe Idiosyncratic Toxicities	Interactions
Carbamazepine	Partial	400-1600+ mg (BID-QID)	4-12+	Diplopia, dizziness, ataxia, hyponatremia	Yes	Bidirectional; (AEDs, OC, AC, many)
Ethosuximide	Absence	500-1500+ mg (BID)	40-100+	Nausea. sedation	Yes	Unidirectional
Phenobarbital	Partial	90-180+ mg (qD)	15-40	Sedation, Psychomotor slowing	Yes	Bidirectional (AEDs, OC, AC, many)
Phenytoin	Partial	200-400+ mg (qD-BID)	8-20+	Sedation, dizziness, ataxia, gingival hyperplasia	Yes	Bidirectional (AEDs, OC, AC, many)
Primidone	Partial	500-1500+ mg (BID-TID)	5-12 (measure phenobarbital)	Sedation, psychomotor slowing	Yes	Bidirectional (AEDs, OC, AC, many)
Valproate	Broad	750-2500+ mg (qD-TID)	50-100+	Nausea, tremor, hair loss, weight gain	Yes	Bidirectional (AEDs)
**Newer AEDs**						
Felbamate	Broad	1800-4800+ mg (BID-TID)	30-100+	Irritability, insomnia, weight loss	Yes	Bidirectional (AEDs, OC, AC)
Gabapentin	Partial	900-3600+ mg (TID-QID)	4-20++	Sedation, dizziness, weight gain	No	None
Lacosamide	Partial, potentially broad	200-600+ mg	?	Sedation, fatigue	?	None known
Lamotrigine	Broad	300-600+ mg (qD-BID)	4-20+	Dizziness, rash	Yes	Bidirectional (AEDs, OC)
Levetiracetam	Broad	1000-3000++ mg (BID)	5-40++	Sedation, dizziness	No	None
Oxcarbazepine	Partial	600-3600+ mg (BID)	10-40+ (MHD)	Sedation, dizziness hyponatremia	Yes	Bidirectional (AEDs, OC)
Rufinamide	Broad	400-3200+ mg daily	?	Sedation, diarrhea	?	Bidirectional
Tiagabine	Partial	16-64 mg (BID-TID)	100-300 ng/mL	Sedation, dizziness	No	Unidirectional
Topiramate	Broad	100-600+ mg (qD-BID)	10-20+	Sedation, cognitive complaints, paresthesias, weight loss, rare nephrolithiasis	No	Bidirectional(AEDs, OC at high doses)
Zonisamide	Broad	100-600+ mg (qD-BID)	10-40+	Sedation, paresthesias, weight loss, rare nephrolithiasis	Yes	Unidirectional

AED = antiepileptic drug;

uG/mL= micrograms/milliliter;

+ = higher doses/levels often additionally effective, as tolerated;

++ = considerably higher doses/levels sometimes additionally effective in intractable patients, as tolerated;

MHD = 10, 11; Monohydroxyderivative active metabolite of oxcarbazepine;

nG/mL = nanograms/milliliter.  Interactions: Unidirectional indicates that other AEDs or drugs may affect this AED;

Bidirectional indicates that other drugs may affect this AED, and this AED affects other drugs;

OC=oral contraceptives, AC=anticoagulants;

many=many other non-AEDs.

**Table 2. Adverse Events Profile.** The instructions to patient for the Adverse Events Profile are as follows: During the past four weeks, have you had any of the problems or adverse effects listed below?  For each item, if it always or often has been a problem, circle 4; if it sometimes has been a problem, circle 3; and so on.  Please answer ever item.

	Always/Often	Sometimes	Rarely	Never
Unsteadiness	4	3	2	1
Tiredness	4	3	2	1
Restlessness	4	3	2	1
Feelings of aggression	4	3	2	1
Nervousness and/or aggression	4	3	2	1
Headache	4	3	2	1
Hair loss	4	3	2	1
Problems with skin (eg acne, rash)	4	3	2	1
Double or blurred vision	4	3	2	1
Upset stomach	4	3	2	1
Difficulty in concentration	4	3	2	1
Trouble with mouth or gums	4	3	2	1
Shaky hands	4	3	2	1
Weight gain	4	3	2	1
Dizziness	4	3	2	1
Sleepiness	4	3	2	1
Depression	4	3	2	1
Memory problems	4	3	2	1
Disturbed sleep	4	3	2	1
