# ERRATUM 2

**DOI:** 10.2174/157015910790909449

**Published:** 2010-03

This is with reference to the article entitled, “Minimizing AED Adverse Effects: Improving Quality of Life in the Interictal State in Epilepsy Care.”, by Dr. St. Louis EK, published in *Current Neuropharmacology*, 106-114 June **2009**, Vol. *7*, No. *2*, pp. 106-114.

Due to an oversight, First name author was cited as Louis EK and now it should read as St. Louis EK.

